# Currarino syndrome associated with an isolated 7q terminal deletion in Korea: a case report

**DOI:** 10.12701/jyms.2026.43.8

**Published:** 2026-01-05

**Authors:** Jin Hee Jung, Juiee Jeong, Sung Hyun Kim

**Affiliations:** Department of Physical Medicine and Rehabilitation, Bundang Jesaeng Hospital, Seongnam, Korea

**Keywords:** Chromosome deletion, Currarino syndrome, Haploinsufficiency

## Abstract

The 7q terminal deletion syndrome is a rare genetic disorder caused by the deletion of the long arm of chromosome 7 between 7q32 and 7q36.3. It is characterized by various clinical symptoms, such as abnormal facial features and impaired mental and physical development. Currarino syndrome is defined by a triad of sacral bone defects, anorectal malformations, and presacral masses and is often associated with mutations in the *MNX1* gene located at 7q36.3. Only a few cases of 7q terminal deletion syndrome have been reported in Korea. In one of these familial cases, Currarino syndrome was associated with a complex chromosomal rearrangement involving a 7q deletion and an 8q duplication. However, to our knowledge, cases of isolated 7q terminal deletions without other structural chromosomal abnormalities have not been described in the literature. We report the case of a 9-month-old girl who presented with the complete Currarino syndrome triad and a 7q35 to 7q36.3 (12 Mb) deletion identified by chromosomal microarray analysis. To the best of our knowledge, this is the first Korean case report of the Currarino triad caused by an isolated terminal 7q deletion.

## Introduction

The 7q terminal deletion syndrome was first characterized by Harris et al. [1] and encompasses a spectrum of clinical phenotypes caused by deletions in the long arm of chromosome 7 [1,2]. The common features of 7q terminal deletion syndrome are developmental delay, intellectual disability, and craniofacial abnormalities [1,3]. Although 7q36 deletions are often associated with holoprosencephaly because of Sonic Hedgehog (*SHH*) gene involvement [2], these deletions can also encompass the motor neuron and pancreas homeobox 1 (*MNX1*) gene, leading to distinct presentations. Only a few cases of isolated heterozygous chromosome 7q terminal deletions between 7q32 and 7q36.3 have been reported [3-7]. Most cases involve 7q36 microdeletions, and this genomic region contains approximately 40 Online Mendelian Inheritance in Man database genes, including *SHH*, engrailed-2 (*EN2*), enhancer of zeste 2 (*EZH2*), limb development membrane protein 1 (*LMBR1*), and *MNX1*. This genomic region may be dosage-sensitive, with key genes of clinical value for identifying and treating this disorder [4,8]. Variations and mutations within a gene can result in various phenotypic expressions, and *MNX1* gene mutations have been linked to Currarino syndrome (CS) [9-11]. CS is defined as a clinical triad of presacral masses, anorectal malformations, and sacral bone deformity [12]. In Korea, previous reports of CS associated with 7q deletions involved complex rearrangements such as concurrent 8q duplications [7]. To the best of our knowledge, no case of an isolated 7q terminal deletion has been documented in Korean literature. Herein, we report the first Korean case of the CS triad caused by an isolated 7q terminal deletion.

## Case

**Ethics statement:** This study was approved by the Institutional Review Board (IRB) of Bundang Jesaeng Hospital (IRB No: Bundang Jesaeng 2025-09-010). Informed consent for the publication of the patient’s clinical details and images was obtained from the patient’s guardian.

A 9-month-old girl was referred to the Department of Rehabilitation Medicine of our hospital on August 2, 2021, for intensive rehabilitation because of global developmental delay. She was born via cesarean section at 38 weeks and 3 days of gestation with a birth weight of 2.96 kg. Her family and perinatal histories were unremarkable.

At birth, she presented with multiple congenital anomalies, including micrognathia, microcephaly, frontal bossing, a short neck, a high-arched palate, and narrow nostrils, which prompted transfer to a tertiary hospital for further evaluation.

**Fig. 1.**
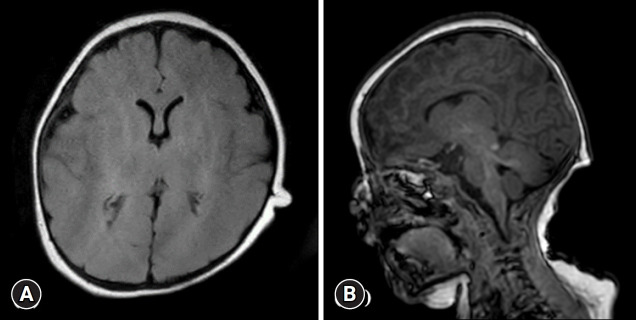
Brain magnetic resonance imaging of the patient. (A) Axial T2-weighted image demonstrates normal cerebral hemispheres and ventricular system. (B) Sagittal T1-weighted image confirms the presence of a normal corpus callosum and other midline structures. No features of holoprosencephaly, ischemic lesion, or hemorrhage are observed.

**Fig. 2.**
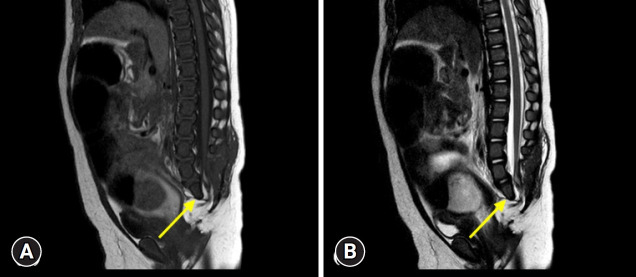
Sagittal magnetic resonance imaging of the lumbosacral spine. T1-weighted (A) and T2-weighted images (B) show a hypoplastic S2 vertebra (yellow arrows) as well as agenesis of the lower sacrum and coccyx.

The initial workup, which included imaging studies, revealed a presacral mass, sacral agenesis, and rectal stenosis, confirming the presence of the CS triad. Chromosomal microarray analysis revealed a 7q35 to 7q36.3 (12 Mb) deletion. Additionally, a next-generation sequencing panel identified several variants of uncertain significance in other genes such as lysine demethylase 6A (*KDM6A*) and SET domain containing 2 (*SETD2*). Brain magnetic resonance imaging (MRI) performed on November 30, 2020, revealed no structural abnormalities (Fig. 1). During early infancy, the patient underwent a series of surgical interventions, including a laparoscopy-assisted colostomy in November 2020, colostomy repair in January 2021, and detethering of the lipomyelomeningocele on March 4, 2021. Lumbosacral spine MRI performed on April 2, 2021, after the surgeries, demonstrated a hypoplastic S2 vertebra and agenesis of the lower sacrum and coccyx (Fig. 2).

Her first developmental assessment at 7 months of age using the Bayley Scales of Infant Development-III showed no delay. However, the follow-up assessments at 17 and 22 months revealed developmental delays in all domains (Table 1). The Sequenced Language Scale for Infants score at 22 months of age indicated a language delay equivalent to that of a 9-month-old infant (Table 2).

The patient underwent continuous intensive outpatient rehabilitation including physical and occupational therapy. The follow-up evaluation at 37 months showed persistent delays across all areas, with only a slight improvement in the cognitive domain, suggesting that substantial catch-up had not occurred (Tables 1, 2).

## Discussion

To the best of our knowledge, this is the first report in Korea of a patient with the complete CS triad caused by an isolated 7q terminal deletion. 7q terminal deletion syndrome was first characterized by Harris et al. [1] in 1977 and presents with characteristics such as growth retardation, intellectual disability, and atypical facial features [1,3]. A familial case of the Currarino triad associated with a complex chromosomal rearrangement involving 7q36.1 terminal deletion and 8q24.3 duplication was previously reported in Korea; however, a case with a pure 7q terminal deletion has not been described to our knowledge [7]. Here, we demonstrate how a specific genetic deletion leads to the clinical features of this rare syndrome.

The 7q36 region contains several dose-sensitive genes critical for embryonic development, such as *SHH*, *EN2*, *EZH2*, and *MNX1* [8]. *LMBR1*, located at 7q36.3, contains a long-range enhancer of the *SHH* gene known as the zone of polarizing activity regulatory sequence (ZRS) [13]. Deletions of *SHH* or its regulatory elements, such as ZRS, are associated with holoprosencephaly [2,4]. However, the brain MRI of our patient was unremarkable, even though the deletion occurred in a critical region known to contain the *SHH* gene. This finding demonstrates the variable penetrance of holoprosencephaly as brain malformations do not manifest in all patients with deletions in this area [4].

Pathogenic variants of *MNX1* are the major genetic causes of CS, a syndrome first linked to the 7q36 region [6,10]. *MNX1* is a key transcription factor in the development of motor neurons and controls functions, such as movement and breathing [14]. However, *MNX1* variants were not detected in our patient [9,11,15]. A comprehensive genetic review revealed that *MNX1* mutations are identified in approximately 57% of patients with CS, with a lower detection rate in sporadic cases than in familial ones [9]. This genetic heterogeneity suggests that the phenotype does not rely solely on coding mutations. In cases of 7q terminal deletions, the most likely pathogenic mechanism is haploinsufficiency of *MNX1*, in which the loss of a single functional allele is insufficient for normal development [9,11]. Furthermore, 7q terminal deletions may disrupt critical regulatory elements within the 7q36 region that are essential for *MNX1* expression, leading to the complete Currarino triad phenotype, even in the absence of coding variants.

Our patient met the diagnostic criteria for complete CS, as first described by Currarino et al. [12]. She presented with a sacral defect, anorectal malformation, and presacral mass [12,16]. The patient was diagnosed with a lipomyelomeningocele. This finding is consistent with CS because the presacral mass in this condition presents with various pathologies, such as lipomyelomeningocele, anterior meningocele, and teratoma [17]. The *KDM6A* and *SETD2* variants that were also detected were considered incidental. Therefore, the complete CS triad observed in our patient was most directly attributable to haploinsufficiency of genes within the isolated 7q36 deletion. In contrast to a reported Korean case of a complex chromosomal rearrangement [7], our patient presented with an isolated 7q deletion, allowing for a more direct correlation between the genetic findings and development of the CS triad.

The patient experienced a persistent global developmental delay despite intensive rehabilitation, a common feature of 7q terminal deletion syndrome [1,8]. This highlights the need for long-term multidisciplinary follow-up involving continuous rehabilitation and developmental assessments.

In conclusion, we reported the first case of CS associated with an isolated 7q terminal deletion in Korea. This case expands the clinical spectrum of this rare condition and is genetically distinct from other 7q deletions previously reported in Korea [7,18].

## Figures and Tables

**Table 1.** Serial assessment results using the Bayley Scales of Infant Development-III

Domain	7 months^b)^	17 months^b)^	22 months^b)^	37 months^b)^
Cognition^a)^	75	55	55	55
Language^a)^	94	64	61	56
Motor^a)^	73	49	52	49

a) Values are reported as composite scores (mean, 100; standard deviation, 15). Scores below 70 indicate delayed development.

b) Chronological age of patient at time of testing.

**Table 2.** Serial assessment results using the Sequenced Language Scale for Infants

Domain	22 months^b)^	27 months^b)^
Receptive^a)^	9 months	9 months
Expressive^a)^	9 months	10 months

a) Values are presented as equivalent age.

b) Chronological age of patient at time of testing.
